# Alcohol consumption and serum uric acid are synergistically associated with renal dysfunction among community‐dwelling persons

**DOI:** 10.1002/jcla.23812

**Published:** 2021-05-07

**Authors:** Ryuichi Kawamoto, Asuka Kikuchi, Taichi Akase, Daisuke Ninomiya, Yoshio Tokumoto, Teru Kumagi

**Affiliations:** ^1^ Department of Community Medicine Ehime University Graduate School of Medicine Toon Japan; ^2^ Department of Internal Medicine Seiyo Municipal Nomura Hospital Seiyo Japan

**Keywords:** alcohol consumption, eGFR, interactive effects, risk factor, serum uric acid

## Abstract

**Background:**

Serum uric acid (SUA) is a key risk factor contributing to renal failure, a serious public health problem. However, few studies have examined whether the interactive relationship between alcohol consumption and SUA is independently associated with the estimated glomerular filtration rate (eGFR).

**Methods:**

Our sample comprised 742 men aged 69 ± 11 years (mean ± standard deviation) and 977 women aged 69 ± 10 years from a rural area. We cross‐sectionally examined the relationships between the confounding factors of alcohol consumption and SUA with renal function denoted by eGFR estimated using CKD‐EPI (Chronic Kidney Disease Epidemiology Collaboration) equations modified by a Japanese coefficient.

**Results:**

In both genders, eGFR increased with a rise in alcohol consumption. This tendency was more pronounced in participants with hyperuricemia, where SUA was greater than 7.0 mg/dL in men and greater than 6.0 mg/dl in women (men: *F* = 41.98, *p* < 0.001; women: *F* = 41.98, *p* < 0.001). A multiple linear regression analysis showed that alcohol consumption (men: *β* = 0.112, *p* < 0.001; women: *β* = 0.060, *p* = 0.011) and SUA (men: *β* = −0.282, *p* < 0.001; women: *β* = 0.317, *p* < 0.001) were significantly and independently related to eGFR. Further, the interactive relationship between alcohol consumption and SUA (men: *F* = 6.388, *p* < 0.001; women: *F* = 5.368, *p* < 0.001) was a significant and independent indicator of eGFR.

**Conclusions:**

These results suggested that alcohol consumption and SUA were synergistically associated with renal dysfunction among community‐dwelling persons.

## Graphical abstract

In both men and women, serum uric acid level is negatively correlated with eGFR. A covariance analysis showed that the regressions based on participants’ drinking status were significantly different (men: *F* = 5.297, *p* = 0.001; women: *F* = 3.068, *p* = 0.027)

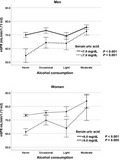

## 1 INTRODUCTION

Serum uric acid (SUA) is one of the major risk factors contributing to renal dysfunction, a severe public health problem. Patients with chronic kidney disease (CKD) generally report high SUA levels. A recent meta‐analysis revealed a direct relationship between elevated baseline SUA levels and incident CKD.^1^ Longitudinal changes in SUA are interactively and independently associated with declining renal function in community‐dwelling older adults.^2^

Several studies have shown a consistent relationship between moderate alcohol consumption and health benefits, including a reduced risk of type 2 diabetes,^3^ coronary heart disease,^4^ ischemic stroke,^5^ cancer mortality in men,^6^ and all‐cause mortality.^7^ Researchers have also investigated the impact of alcohol consumption on various renal disorders. However, the findings on this impact have been inconsistent. A study of 1,658 nurses concluded there was no correlation between alcohol consumption and renal dysfunction.^8^ Examining a large cohort of healthy men, Schaeffner et al. ^9^ ^10^ showed that moderate alcohol consumption was inversely related with the risk of renal dysfunction. Buja et al.^10^ demonstrated a U‐shaped relationship between alcohol consumption and the incidence of renal impairment in women who drink more than 24 g of alcohol per day. Two retrospective analyses showed a correlation between moderate alcohol consumption and increased risk of renal dysfunction ^11^ or end‐stage renal disease.^12^ Further, alcohol consumption leads to hyperuricemia as a result of the high purine content of certain types of alcoholic beverages,^13^ increased urate production from purine nucleotide degradation during ethanol catabolism, and lactic acid inhibition of renal urate excretion.^13^ However, to the best of our knowledge, few studies have examined the interactive effects of alcohol consumption and SUA levels on renal dysfunction in Japanese populations.^14^

This study first examined the relationship between the confounding factors of alcohol consumption and SUA and renal function denoted by the estimated glomerular filtration rate (eGFR). Second, it investigates whether the interactive relationship between alcohol consumption and SUA is independently related to eGFR using cross‐sectional data from community‐dwelling persons.

## 2 MATERIALS AND METHODS

### 2.1 Subjects

This cross‐sectional study was designed as part of a research project conducted by researchers at the Nomura Welfare Center.^15^ Survey participants included individuals who underwent an annual community health examination at the Nomura Welfare Center in a rural region of Ehime Prefecture, Japan. This study excluded all individuals who were on SUA‐lowering drugs or who had a baseline eGFR of less than 10 ml/min/1.73 m^2^. Data on medical history, current status, and medications (eg, antihypertensive, antilipidemic, antidiabetic, and SUA‐lowering medications) were obtained during interviews conducted using a structured questionnaire. The study is in line with the Declaration of Helsinki Ethical Principles, written informed consent was obtained from each subject, and the study was approved by the Ehime University Medical School Ethics Committee (Institutional Review Board: 1903018).

### 2.2 Evaluation of Risk Factors

Data on demographic characteristics and confounding factors were collected from the participants' clinical files. Participants wore light clothing and removed their shoes for the height and body weight measurements. We divided weight (kg) by height squared (m^2^) to calculate body mass index (BMI). Smoking status was defined as the product of cigarette packs smoked per day and the number of years of smoking (packs・years). Participants were categorized as non‐smokers, ex‐smokers, light smokers (<20 packs/year), or heavy smokers (≥20 packs/year). We measured daily alcohol consumption in units of sake equivalent to 22.9 g of ethanol. Accordingly, participants were classified as non‐drinkers, occasional drinkers (<1unit/day), daily light drinkers (1–2 units/day), or daily moderate drinkers (2–3 units/day). We used an appropriately sized cuff around the upper right arm of participants to estimate systolic blood pressure (SBP) and diastolic blood pressure (DBP) and took two estimates with an interval of 5 min. We used the mean of the two consecutive measurements for analysis. Participants were asked to fast overnight before triglyceride (TG), high‐ and low‐density lipoprotein cholesterol (HDL‐C and LDL‐C), SUA, and hemoglobin A1c (HbA1c) levels were measured. We used the CKD‐EPI (Chronic Kidney Disease Epidemiology Collaboration) equation modified with a Japanese coefficient to estimate glomerular filtration ratio (eGFR). The equation for men with creatinine (Cr) ≤0.9 mg/dl is 141 × (Cr/0.9) ^−0.411^ × 0.993 ^age^ × 0.813; for those with Cr >0.9 mg/dl, it is 141 × (Cr/0.9) ^−1.209^ × 0.993 ^age^ × 0.813. For women with Cr ≤0.7 mg/dl, the equation is 144 × (Cr/0.7) ^−0.329^ × 0.993 ^age^ × 0.813; for those with Cr >0.7 mg/dl, it is 144 × (Cr/0.7) ^−1.209^ × 0.993 ^age^ × 0.813.^16^ Hyperuricemia was defined as SUA levels greater than 7.0 mg/dl for men or 6.0 mg/dl for women.^17^ CKD was determined by the presence of dipstick‐positive proteinuria (≥1+) or a low eGFR (<60 ml/min/1.73 m^2^).^18^ Cardiovascular diseases (CVD) included ischemic heart disease, ischemic stroke, and peripheral vascular disease.

### 2.3 Statistical analysis

We performed statistical analyses using SPSS Statistics version 26 for Windows (IBM Japan). If data were normally distributed, continuous variables were denoted as mean ± standard deviation (SD); if not (eg, for TG and HbA1c), the variables were represented as median (interquartile range) values. Parameters with non‐normal distributions were analyzed following log‐transformation. We divided the participants into two groups based on the presence or absence of hyperuricemia. Next, we analyzed the differences in means and prevalence between the two groups by conducting Student's *t* tests on continuous data and χ^2^ tests on categorical data. A multiple linear regression analysis was conducted to examine the impact of all confounding factors (ie, age; BMI; smoking habit; alcohol consumption; exercise regime; history of CVD, SBP, TG, SUA, and LDL‐C and HDL‐C levels; and the use of antihypertensive, antilipidemic, HbA1c, or antidiabetic medication) on eGFR in both genders. Finally, we employed a general linear model to examine the synergistic effect of alcohol consumption (eg, non‐drinkers, occasional drinkers, daily light drinkers, and daily moderate drinkers) and SUA (eg, men, <7.0 mg/dl and ≥7.0 mg/dl; women, <6.0 mg/dl and ≥6.0 mg/dl) on eGFR. A *p*‐value less than 0.05 was considered statistically significant.

## 3 RESULTS

### 3.1 Participants’ background characteristics stratified by gender and hyperuricemia status.

Table 1 presents the background characteristics for men and women based on the presence and absence of hyperuricemia. The participants were 742 men aged 69 ± 11 years and 977 women aged 69 ± 10 years. Men with hyperuricemia reported significantly higher BMI, alcohol consumption, DBP, TG, and SUA than normouricemic men, although those with hyperuricemia had a significantly lower eGFR. Similarly, for the women, those with hyperuricemia showed significantly higher age, BMI, alcohol consumption, SBP, antihypertensive medication use, TG, HbA1c, and SUA than normouricemic women. However, women with hyperuricemia exhibited significantly lower HDL‐C level and eGFR.

**TABLE 1:** Background characteristics of participants stratified by gender and SUA level

Characteristic *N* = 1,719	Men *N* = 742			Women *N* = 977		
	SUA <7.0 mg/dl *N* = 588	SUA ≥7.0 mg/dl *N* = 154	*p*‐value	SUA <6.0 mg/dl *N* = 854	SUA ≥6.0 mg/dl *N* = 123	*p*‐value
Age (years)	69 ± 11	67 ± 12	0.061	69 ± 10	71 ± 9	**0.024**
Body mass index (kg/m^2^)	22.9 ± 3.0	23.7 ± 3.1	**0.004**	22.3 ± 3.2	24.0 ± 3.1	**<0.001**
Smoking habit (non = 0/ex = 1/light = 2/heavy = 3) (%)	43.2/36.4/6.6/13.8	40.3/42.9/5.2/11.7	0.495	96.6/2.2/0.7/0.5	95.9/2.4/1.6/0	0.632
Alcohol consumption (never = 0/occasional = 1/light = 2/moderate=3) (%)	27.9/23.5/16.2/32.5	14.9/23.4/16.2/45.5	**0.003**	72.7/21.4/3.9/2.0	58.5/29.3/8.1/4.1	**0.006**
Exercise habits (%)	36.2	35.7	0.925	37.7	35.8	0.765
Cardiovascular disease (%)	9.0	12.3	0.222	3.9	6.5	0.223
Systolic blood pressure (mm Hg)	135 ± 18	135 ± 15	0.868	135 ± 18	140 ± 14	**0.013**
Diastolic blood pressure (mm Hg)	79 ± 10	81 ± 10	**0.006**	76 ± 10	78 ± 9	0.140
Antihypertensive medication (%)	41.5	45.5	0.410	40.0	61.8	**<0.001**
Triglycerides (mg/dl)	88 (67–127)	99 (70–154)	**<0.001**	82 (64–112)	102 (75–157)	**<0.001**
LDL cholesterol (mg/dl)	115 ± 29	111 ± 30	0.177	125 ± 29	124 ± 33	0.954
HDL cholesterol (mg/dl)	62 ± 16	61 ± 16	0.494	69 ± 17	63 ± 15	**<0.001**
Antilipidemic medication (%)	13.4	9.1	0.172	27.0	39.0	**0.008**
Hemoglobin A1c (%)	5.6 (5.4–6.0)	5.7 (5.4–6.0)	0.260	5.7 (5.4–5.9)	5.9 (5.5–6.2)	**<0.001**
Antidiabetic medication (%)	12.6	13.6	0.786	4.8	8.9	0.081
SUA (mg/dl)	5.5 ± 1.0	7.7 ± 0.6	**<0.001**	4.4 ± 0.9	6.6 ± 0.6	**<0.001**
eGFR (mL/min/1.73 m^2^)	72.4 ± 11.2	66.5 ± 17.1	**<0.001**	74.1 ± 10.1	64.6 ± 15.1	**<0.001**

Note

*p‐values: Student's t test for continuous variables or the *χ* ^2^ ‐test for categorical variables. Bolded numbers indicate significance.

Abbreviations: eGFR, estimated glomerular filtration rate; HDL, high‐density lipoprotein; LDL, low‐density lipoprotein; SUA, serum uric acid. Data are presented as a mean ± standard deviation. Data for triglycerides and hemoglobin A1c were skewed and are presented as median (interquartile range) values and were log‐transformed for analysis.

### 3.2 Relationship between alcohol consumption and eGFR stratified by gender and hyperuricemia status.

**FIGURE 1:**
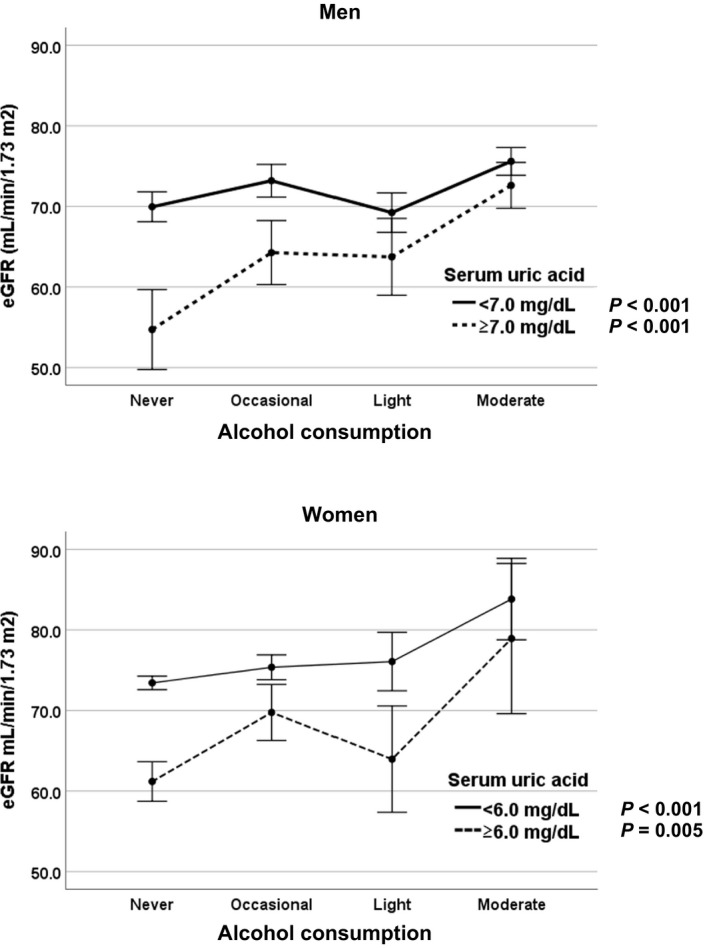
Relationships between alcohol consumption and estimated glomerular filtration rate (eGFR) by gender and drinking status. In both men and women, alcohol consumption is positively correlated with eGFR. A covariance analysis showed that the regressions based on participants’ ​serum uric acid level were significantly different (men: *F* = 5.297, *p* = 0.001; women: *F* = 3.068, *p* = 0.027)

Figure 1 shows that alcohol consumption is positively correlated with eGFR for both genders. The covariance analysis determined that, for both men and women, the relationship between alcohol consumption and eGFR was significantly different for participants with and without hyperuricemia (men: *F* = 5.297, *p* = 0.001 and women: *F* = 3.068, *p* = 0.027).

### 3.3 Relationship between background characteristics and eGFR stratified by gender.

Table 2 presents the findings for the relationships between the confounding factors and eGFR for both genders. According to a multiple linear regression analysis, alcohol consumption and SUA, as well as age, BMI, antihypertensive medication use, and TG, were significantly and independently associated with eGFR.

**TABLE 2:** Relationship between background characteristics and eGFR of participants by gender

Characteristic N = 1,719	eGFR	
	Men *N* = 742 *β* (*p*‐value)	Women *N* = 977 *β* (*p*‐value)
Age	**0.604 (<0.001)**	**−0.588 (<0.001)**
Body mass index	0.044 (0.139)	**0.084 (0.001)**
Smoking habit (non = 0/ex = 1/light = 2/heavy = 3)	0.042 (0.126)	0.018 (0.427)
Alcohol consumption (never = 0/occasiona l= 1/light = 2/moderate = 3) (%)	**0.112 (<0.001)**	**0.060 (0.011)**
Exercise habits (N = 0, Yes = 1)	0.047 (0.078)	0.003 (0.901)
Cardiovascular disease (N = 0, Yes = 1)	0.024 (0.386)	0.003 (0.894)
Systolic blood pressure	0.023 (0.434)	0.026 (0.302)
Antihypertensive medication (N = 0, Yes = 1)	**0.092 (0.002)**	0.031 (0.219)
Triglycerides	0.009 (0.769)	**0.057 (0.034)**
LDL cholesterol	0.005 (0.859)	0.006 (0.813)
HDL cholesterol	0.027 (0.375)	0.023 (0.383)
Antilipidemic medication (N = 0, Yes = 1)	0.035 (0.212)	0.004 (0.859)
Hemoglobin A1c	0.054 (0.080)	0.016 (0.552)
Antidiabetic medication (N = 0, Yes = 1)	0.035 (0.258)	0.018 (0.490)
Serum uric acid	**0.282 (<0.001)**	**0.317 (<0.001)**
R^2^	**0.505 (<0.001)**	**0.523 (<0.001)**

Note

Data for triglycerides and hemoglobin A1c were skewed and are presented as median (interquartile range) values and were log‐transformed for analysis. Bolded numbers indicate significance.

Abbreviatiions: β, standard coefficient; eGFR, estimated glomerular filtration rate.

### 3.4 Interactive effects of alcohol consumption and SUA on eGFR stratified by gender.

In addition to direct associations, this study examined the statistical significance of the interactive relationships using a general linear model with confounding factors (Table 3). The results indicate that the interaction between alcohol consumption and SUA is a significant and independent determinant of eGFR.

**TABLE 3:** Effect of interaction between drinking status and serum uric acid level on the eGFR of participants by gender

Characteristics *N* = 1,719	eGFR	
	Men *N* = 742 *F* (*p*‐value)	Women *N* = 977 *F* (*p*‐value)
Age	**390.2 (<0.001)**	**539.6 (<0.001)**
Body mass index	1.581 (0.209)	**11.08 (0.001)**
Smoking habit (non=0/ex=1/light=2/heavy=3)	2.312 (0.129)	0.624 (0.430)
Alcohol consumption (never = 0/occasional = 1/light = 2/moderate=3) (%)	**4.049 (0.007)**	**3.507 (0.015)**
Exercise habits (N=0, Yes=1)	3.094 (0.079)	0.017 (0.895)
Cardiovascular disease (N=0, Yes=1)	0.613 (0.434)	0.001 (0.970)
Systolic blood pressure	0.744 (0.389)	1.208 (0.272)
Antihypertensive medication (N=0, Yes=1)	**8.275 (0.004)**	1.919 (0.166)
Triglycerides	0.400 (0.527)	**5.129 (0.024)**
LDL cholesterol	0.009 (0.926)	0.173 (0.677)
HDL cholesterol	0.738 (0.390)	1.005 (0.316)
Antilipidemic medication (N=0, Yes=1)	1.742 (0.187)	0.087 (0.768)
Hemoglobin A1c	2.971 (0.085)	0.452 (0.502)
Antidiabetic medication (N=0, Yes=1)	1.391 (0.239)	0.720 (0.396)
Serum uric acid	**111.4 (<0.001)**	**28.77 (<0.001)**
Alcohol consumption *serum uric acid	**6.388 (<0.001)**	**5.368 (0.001)**

Note

Data for triglycerides and hemoglobin A1c were skewed and were log‐transformed for analysis. Bolded numbers indicate significance.

Abbreviations: eGFR, estimated glomerular filtration rate.

* The net effect of each interaction was estimated using a general linear model.

### 3.5 Adjusted eGFR based on hyperuricemia status, stratified by gender and alcohol consumption.

As shown in Table 4, for both genders, participants with hyperuricemia displayed a higher multiple‐adjusted eGFR with increasing alcohol consumption. In addition, we observed that interaction between alcohol consumption and SUA affects eGFR.

**TABLE 4:** Adjusted eGFR based on serum uric acid level in participants categorized by gender and drinking status

Characteristic	Alcohol consumption				
Men *N* = 742	non‐drinkers *N* = 187	Occasional (<1 unit/day) *N* = 174	Daily light (1–2 units/day) *N* = 120	Daily moderate (2–3 units/day) *N* = 261	
Age‐adjusted eGFR (95% CI)					
Serum uric acid <7.0 mg/dl *N* = 588	71.8 (70.3**–**73.2)	72.5 (70.9**–**74.1)	71.8 (69.9**–**73.8)	74.0 (72.7**–**75.4)	**<0.001**
Serum uric acid ≥7.0 mg/dl *N* = 154	57.7 (53.8**–**61.6)	62.2 (59.1**–**65.3)	64.6 (60.9**–**68.3)	**70.0 (67.8–72.3)** ^a^, ^d^	
Multiple‐adjusted eGFR (95% CI)					
Serum uric acid <7.0 mg/dl *N* = 588	71.9 (70.4**–**73.4)	72.4 (70.8**–**73.9)	71.7 (69.8**–**73.6)	73.8 (72.4**–**75.1)	**0.001**
Serum uric acid ≥7.0 mg/dL *N* = 154	58.7 (54.8**–**62.5)	63.0 (59.9**–**66.1)	65.5 (61.9**–**69.2)	**69.9 (67.6–72.2)** ^a^, ^d^	

Women *N* = 977	non‐drinkers *N* = 693	Occasional (<1 unit/day) *N* =219	Light (1–2 units/day) *N* = 43	Moderate (2–3 units/day) *N* = 22	*p*‐value for interaction
Age‐adjusted eGFR (95% CI)					
Serum uric acid <6.0 mg/dL *N* = 854	73.9 (73.3**–**74.5)	74.0 (72.8**–**75.2)	74.0 (71.2**–**76.8)	75.9 (71.9**–**79.8)	**0.005**
Serum uric acid ≥6.0 mg/dL *N* = 123	63.8 (61.9**–**65.7)	**69.4 (66.8–72.1)** ^b^	64.9 (59.9**–**70.0)	**75.1 (67.9–82.2)** ^c^	
Multiple‐adjusted eGFR (95% CI)					
Serum uric acid <6.0 mg/dL *N* = 854	73.9 (73.2**–**74.5)	73.9 (72.7**–**75.1)	74.0 (71.2**–**76.8)	75.9 (71.9**–**80.0)	**0.004**
Serum uric acid ≥6.0 mg/dL *N* = 123	64.0 (62.1**–**65.9)	**69.4 (66.7–72.1)** ^b^	65.2 (60.1**–**70.3)	**76.6 (69.3–83.8)** ^b^	

Note

Multiple‐adjusted odds ratio for all confounding factors listed in Table 2. Data for triglycerides and hemoglobin A1c were skewed and were log‐transformed for analysis. Numbers in bold indicate significance.

Abbreviations: CI, confidence interval; eGFR, estimated glomerular filtration rate.

^a^ *p* < 0.001

^b^ *p* < 0.01

^c^ *p* < 0.05 versus never drinkers

^d^ *p* < 0.005 versus occasional drinkers.

## 4 DISCUSSION

This study examined the association of alcohol consumption and SUA with renal function (ie, eGFR) in the general population. The findings reveal a relationship between various confounding factors and eGFR, which is consistent with previous research.^19^ More specifically, this study demonstrates that age, alcohol consumption, antihypertensive medication, TG, and SUA all have a significant relationship with eGFR. In addition to these direct associations, the interaction between alcohol consumption and SUA was found to be a significant and independent determinant of eGFR. This research, to the best of our knowledge, is the first to demonstrate that alcohol consumption and SUA have an interactive effect on renal dysfunction, and that alcohol consumption modifies the relationship between SUA and renal dysfunction.

About 20.8% of men reported having hyperuricemia, compared to 12.6% of women, which is consistent with results of previous studies.^20^ ^21^ The difference can be explained by the presence of estrogen in women, which increases uric acid excretion.^22^ Hyperuricemia is an independent risk factor contributing to renal dysfunctions such as microalbuminuria ^23^ and CKD ^24^ ^25^.^26^ ^27^ However, studies have shown that increased SUA is a consequence of coexisting risk factors such as hypertension, obesity, dyslipidemia, and insulin resistance.^28^ Nevertheless, a growing body of research suggests hyperuricemia is a marker for future renal dysfunction, rather than the declining renal excretion of uric acid.^1^ Recent studies have explained several mechanisms behind the causal relationship between hyperuricemia and CKD. These include insulin resistance,^29^ increased synthesis of interleukin‐6, activation of the local renin‐angiotensin system (RAS), proinflammatory and proliferative actions, impaired endothelial nitric oxide production,^30^ higher oxidative stress, endothelial dysfunction,^31^ and vascular smooth muscle cell proliferation.^32^

Studies have associated alcohol consumption with aggravated CKD ^12^ ^33^ or increased all‐cause mortality in patients with CKD.^34^ Numerous experimental studies have confirmed that alcohol consumption damages the glomeruli and renal tubules, leading to albuminuria and reduced GFR, although some research has determined the opposite.^35^ Alcohol consumption increases the production of reactive oxygen species (ROS), and this contributes to lipid peroxidation and damages antioxidant capacity.^36^ The long‐term consumption of alcohol activates the RAS and increases sympathetic nervous system activity, which elevates the SBP and damages the normal structure of the glomeruli.^37^ These factors potentially cause renal injury through hemodynamic disorders and inflammation.^38^ However, some clinical studies have shown an association between moderate alcohol consumption and a reduced incidence of CKD ^39^ and end‐stage renal disease.^40^ That is, moderate alcohol consumption lowers the risk of type 2 diabetes and curbs rises in HDL‐C, which are both closely related to CKD,^35^ thus reducing the decline in renal function. Another explanation is the lowered risk of CVD, a key contributing factor to the majority of deaths among patients with CKD.^41^ While these inconsistent findings can be attributed to varying clinical research designs ^42^ and the interactive effects of alcohol consumption and SUA on eGFR, exact explanations remain unclear.

This study is subject to several important limitations. First, we used a cross‐sectional design and were unable to establish causality. Further, we assessed alcohol consumption, SUA, and renal function at a single time point and did not conduct follow‐up evaluations to determine clinical impact. Second, compared to direct estimations of renal function, single assessments of serum creatinine produce a rather imprecise eGFR. Finally, some of the study population reported several risk factors (eg, advanced age, hypertension, dyslipidemia, and diabetes) and the possible effects of the underlying diseases and medications could not be excluded from the present findings.

In conclusion, this cross‐sectional study highlights the possibility that a moderate consumption of alcohol is not related to an increased risk of renal dysfunction in either gender. In fact, it shows that moderate alcohol consumption is inversely related with renal dysfunction. This indicates that alcohol consumption and SUA were synergistically associated with renal dysfunction among community‐dwelling persons. The mechanisms underlying this association warrant further research.

## CONFLICT OF INTEREST

The authors declare that they have no competing interests.

## AUTHOR CONTRIBUTIONS

RK participated in the design of the study, performed the statistical analysis, and drafted the manuscript. RK AK, TA, DN, YT, and TK contributed to the acquisition and interpretation of data. RK, DN, and TK contributed to the conception and design of the statistical analysis. All authors read and approved the manuscript.

## ETHICAL APPROVAL

All procedures performed in studies involving human participants were in accordance with the ethical standards of the institutional research committee at which the studies conducted (Institutional Review Board: 1903018).

## Data Availability

The datasets analyzed in this study are available from the corresponding author (Ryuichi Kawamoto, rykawamo@m.ehime-u.ac.jp) on reasonable request.
